# Supplementary material for: Decomposing virulence to understand bacterial clearance in persistent infections
Source: Nat Commun. 2022 Aug 26;13:5023. doi: 10.1038/s41467-022-32118-1 (PMC9418333; doi:10.1038/s41467-022-32118-1)
Supplement: Supplementary file 1 — Supplementary Information [file 41467_2022_32118_MOESM1_ESM.pdf]

Supplementary Information

Acuña Hidalgo, Silva, Franz, Regoes & Armitage

Decomposing virulence to understand bacterial clearance in persistent infections

Supplementary Note

Supplementary legend information for Fig. 2: Effects of PPP and exploitation on optimal host clearance effort

In our first two hypotheses (H1 and H2) we assumed that changes in PPP and exploitation lead to changes in the optimal host clearance effort. In contrast, in our third hypothesis (H3) we assumed that changes in exploitation do not necessarily affect the optimal host clearance effort. In the following we explain in more detail how we derived these contrasting assumptions.

A key assumption relates to how survival benefits (Fig. 2a, b) and cost (Fig. 2c, d) are combined to generate total host survival (Fig. 2e). We assumed that the functions that describe the separate effects on host survival (Fig. 2b, d) are multiplied to generate their combined effect (Fig. 2e). In our hypotheses, we assume that PPP and exploitation do not affect the relationship between host clearance effort and costs (Fig. 2c, d). Instead, PPP and exploitation are assumed to only change survival benefits (Fig 2a, b). Importantly, these changes differ qualitatively between H1 and H2 on the one side and H3 on the other side – and these qualitative differences are the reason for the assumed effects on optimal host clearance effort.

In our third hypothesis we focussed on the effect that increased exploitation should make it more difficult to clear an infection, which would decrease the clearance rate for any given host clearance effort (Fig. 2k). As a consequence, for any given host clearance effort the host survival rate should also decrease (Fig. 2l). Importantly, we assumed that this reduction in host survival should be more or less proportional, i.e., for any given host clearance effort, host survival should decrease more or less by the same factor x – without changing that shape of the functional relationship between host clearance effort and host survival (Fig. 2l). Accordingly, because of the multiplicative effect on total host survival, there is again only a proportional difference between different exploitation scenarios (Fig. 2m). Specifically, total host survival should decrease by the same factor x – again without affecting the shape for the functional relationship between host clearance effort and total host survival (Fig. 2m). If the shape of this functional relationship stays the same, then this implies that also the optimal host clearance effort stays the same across different levels of exploitation.

In our first and second hypotheses we focussed on the effect that increased PPP and exploitation increase pathogen virulence, which reduces host survival (Fig. 2h). Importantly, the related effects on survival benefits should strongly differ to the effects described above for H3. Specifically, we assume that changes in host survival are far from being proportional (Fig. 2h). Instead, an increase in PPP or exploitation should have a much weaker effect when the host clearance effort is large. This assumption is based on the reasoning that a higher host clearance effort leads to faster clearance, which means that the infection length is shorter. In turn, a shorter infection length implies that changes in virulence (which are caused by changes in PPP and exploitation) have smaller impacts on host survival. Overall, this leads to disproportionate changes in host survival, with a much stronger reduction of benefits for smaller host clearance efforts (Fig. 2h). Accordingly, there is also a much stronger reduction in the total host survival for smaller host clearance efforts (Fig. 2i). This disproportionate reduction is the reason for the assumed increase in optimal host clearance effort (Fig. 2i). Finally, we note that the functional relationships depicted in Fig. 2 were chosen to depict the basic effects of host clearance effort on survival costs and benefits and how PPP and

exploitation could affect these relationships. We do not want to imply that the actual functional relationships between these quantities are fully captured by these illustrations.

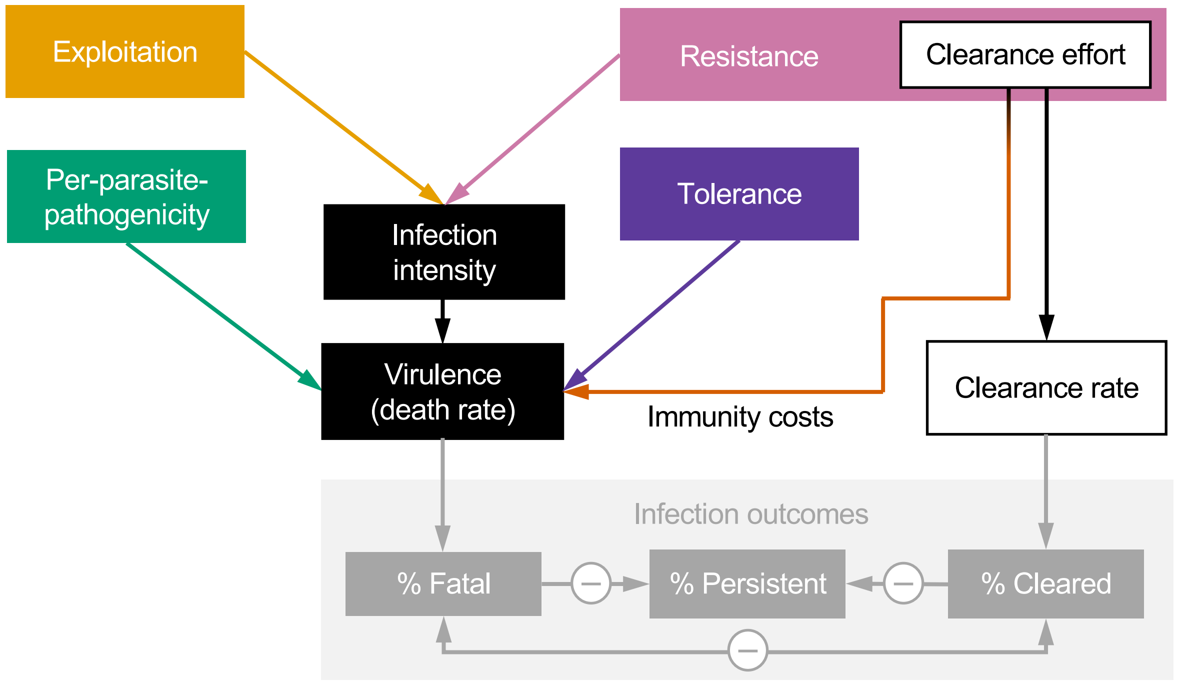

Supplementary Figure 1. Schematic for how pathogen factors (exploitation and per parasite pathogenicity) and host factors (resistance and tolerance) contribute to virulence. This scheme contains all factors (in coloured and black boxes) included in the framework proposed by Råberg and Stjernmann [1], and we extend it by including the terms in the white and grey boxes. "Resistance" can be described as the ability to limit/reduce pathogen burden, and it involves directly fighting against the pathogen [2,3]. Here host "Clearance effort" is considered as a component of host resistance, and "Clearance rate" as a consequence thereof. Host "Clearance effort" is conceptualized as the strength of the resistance mechanisms that act towards pathogen clearance. The "Clearance rate" determines the probability of clearing an infection, and it thereby links to the percentage of hosts in the population that have cleared an infection. We note that tolerance acts to ameliorate immunity costs and also other costs of infection, e.g., pathogen virulence factors, but that for simplicity these effects are not illustrated above. We also note that immunity costs can affect reproduction, and thereby fecundity tolerance.

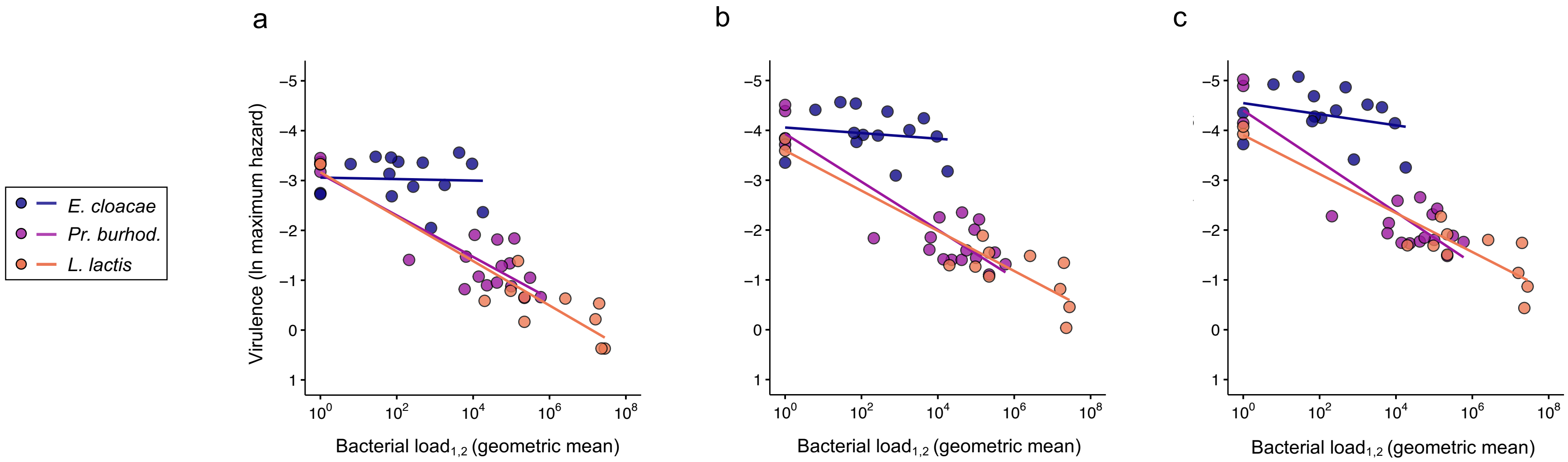

	df	F	P	df	F	P	df	F	P
Bacterial species	2,39	0.047	0.95	2,39	0.71	0.50	2,39	1.34	0.27
Bacterial load	1,39	0.027	0.87	1,39	0.34	0.56	1,39	1.30	0.26
Bacterial species × bacterial load	2,39	8.92	0.00065	2,39	7.29	0.0020	2,39	6.02	0.0053

Supplementary Figure 2. Per parasite pathogenicity using different smoothing parameter values to estimate the maximum hazard. Per parasite pathogenicity is given as the relationship between bacterial load and maximum hazard. The bacterial load data is the same as that given in Fig 5a, with the addition of the Ringer's treatment control. The maximum hazard data is estimated from survival data for the corresponding injection doses and experimental replicates. Maximum hazard is plotted as the inverse, such that the hazard (virulence) increases with proximity to the x-axis. The maximum hazard was estimated from time to death data using four different values (1, 2, 3 and 5) for the smoothing parameter, *b,* as specified using "bw.grid". Shown above are a. *b* = 1, b. *b* = 3, c. *b* = 5 *(b = 2* is shown in Fig. 5b). Coloured lines show the linear regressions. The data were analysed using linear models (see statistical analyses section of methods, Model 2). Tests were two-tailed. The corresponding statistical results are shown below each panel, where maximum hazard was the dependent variable.

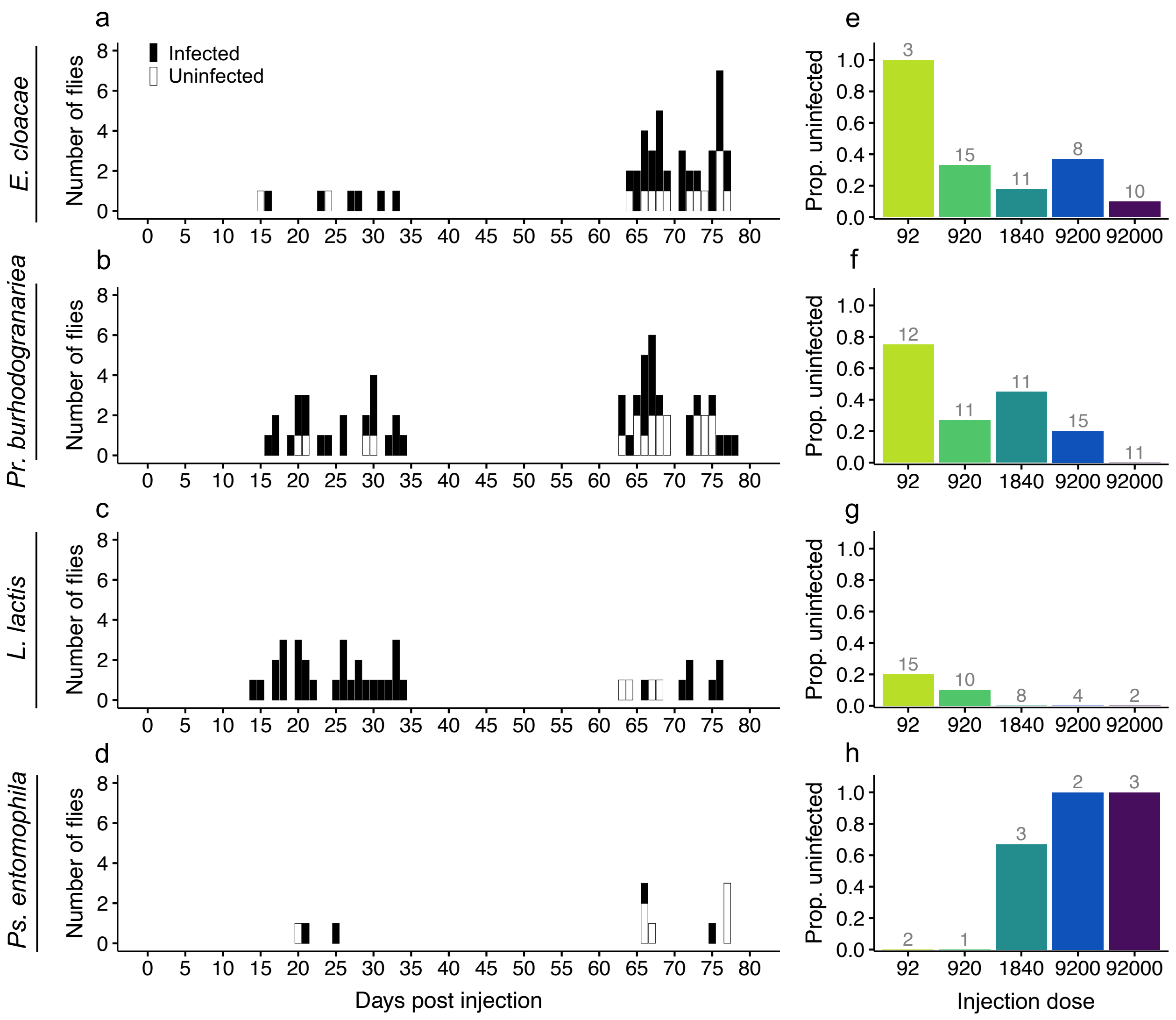

Supplementary Figure 3. Bacterial clearance in dead flies. Each row shows flies that had been injected with one of four bacterial species. a-d The number of dead flies that were infected and uninfected according to the day post injection at which they died and were homogenised. Dead flies were homogenised at between 14 and 35, and 56 and 78, days post injection. e-h The same data as shown in the left-hand panels but graphed by injection dose. Numbers above the bars indicate the total numbers of flies from which the proportions were calculated, *i.e.*, the total numbers of flies homogenised. Note that we cannot distinguish between flies that had cleared the infection and those where the bacterial load was below our detection limit (see methods). Summing up across all doses and days, 29.8 % (14 out of 47) of *E. cloacae*-injected flies, 33.3 % (20 out of 60) of *Pr. burhodogranariea*-injected flies, 10.3 % (4 out of 39) of *L. lactis*-injected flies, and 66.7 % (8 out of 12) of *Ps. entomophila*-injected flies cleared the infection before death.

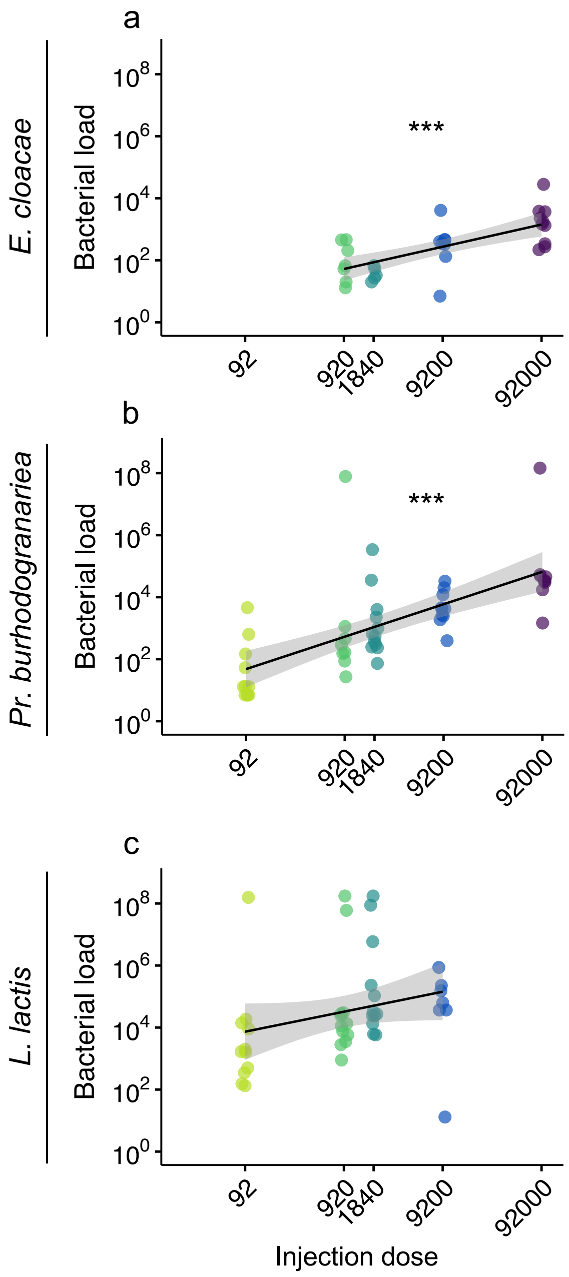

Supplementary Figure 4. The relationship between bacterial load at seven days post injection, and the initial injection doses. Each row shows data from one bacterial species. Panel a contains no flies injected with 92 CFUs because all flies had a bacterial load of zero at day seven; c contains no flies injected with 92,000 CFUs because all flies had died by this time point. Each circle is the bacterial load of one fly, they are jittered along the x-axis to aid visualisation of overlapping data points, and they are coloured according to the injection dose. Flies with zero bacterial load are not shown (see methods). Point estimates of the effect of injection dose on bacterial load are shown in black with corresponding 95 % confidence intervals. The data were analysed using linear models (see statistical analyses section of methods, Model 4, and Supplementary Table 5). Tests were two-tailed. Asterisks denote significant correlations, where $p < 0.0001$. For *L. lactis* $p = 0.058$ – see main text.

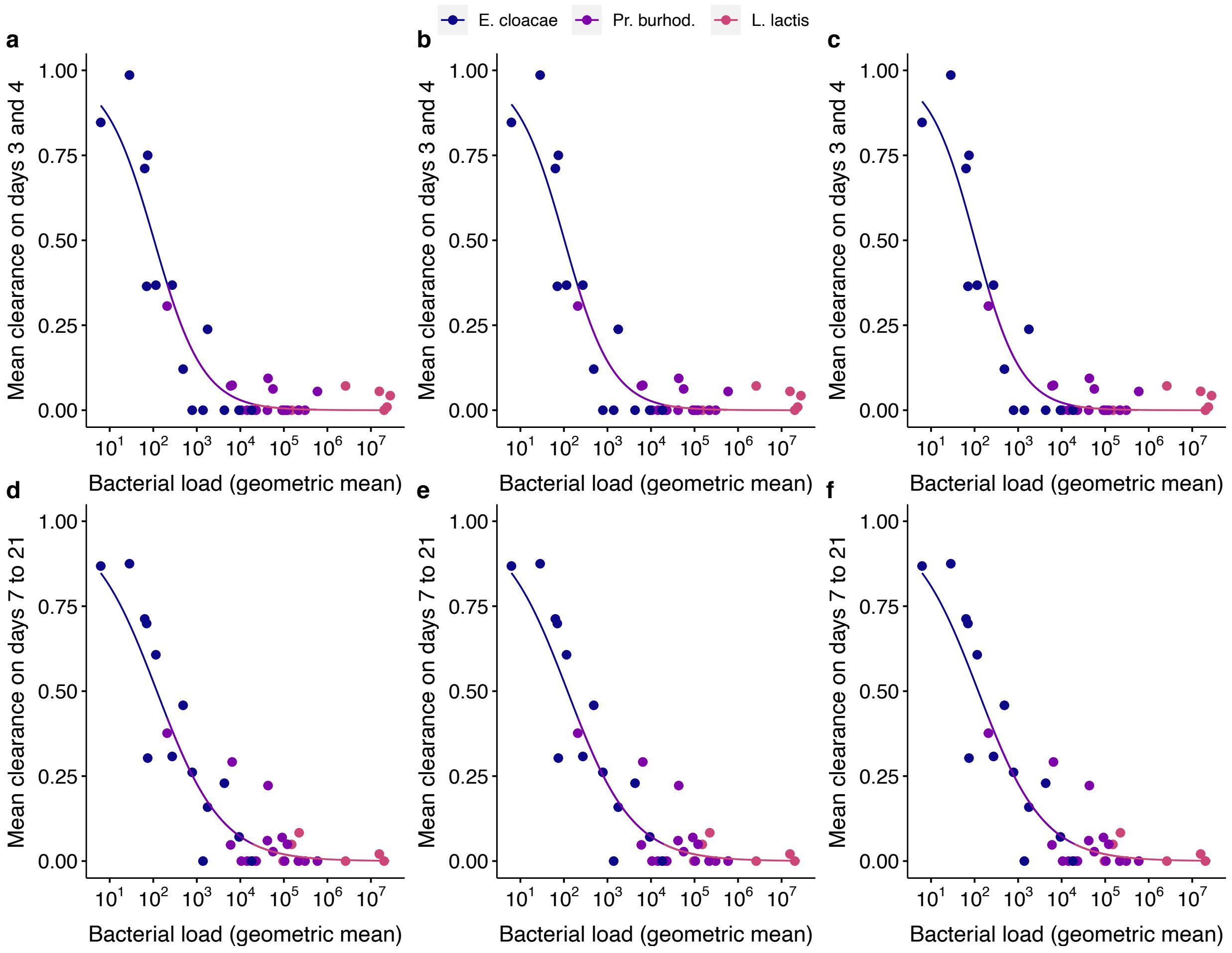

Supplementary Figure 5. Effect of exploitation (bacterial load) on clearance for different values of the smoothing parameter *b* in the estimation of maximum hazard that is used for the calculation of PPP: *b* = 1 (a, d), *b* = 3 (b, e), *b* = 5 (c, f). The geometric mean of bacterial load was calculated from days 1 and 2 post injection. Each data point is from one injection dose per bacteria, per experimental replicate, and gives the mean proportion of cleared infections on days three and four (a-c) and days 7 to 21 (d-f). The key for the bacterial species is shown at the top.

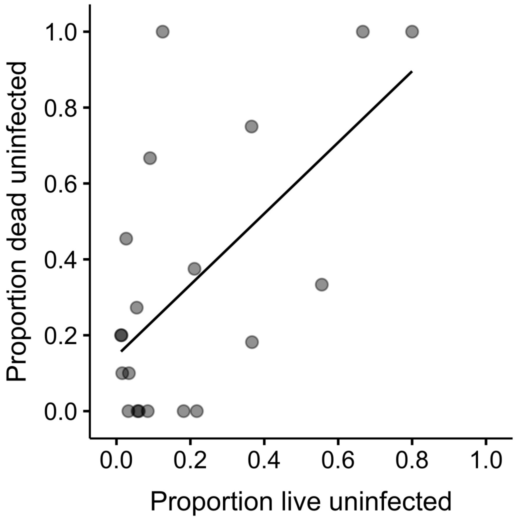

Supplementary Figure 6. Proportion of live and dead flies that were uninfected across bacterial species and doses. Each data point is the proportion for one bacterial species and dose. Darker points are due to overlapping data. Despite variation in the time post infection at which live and dead flies were sampled, across bacterial species and doses, the proportion of living flies that cleared an infection was a predictor for the proportion of dead flies that cleared an infection, i.e., There is a significant relationship between the two variables (two-tailed test: LR Chi Square = 7.11, df = 2,17, p = 0.0285). Most of the data points lie above, rather than on, the diagonal (i.e., higher clearance in dead flies) possibly because the dead flies were on average homogenised later in the infection, giving time for more clearance to take place before being sampled.

Supplementary Table 1. Pairwise comparisons between bacterial species for differences in virulence. Virulence is measured as maximum hazard (Model 1). The "emmeans"[4] statistical package in R was used to perform multiple comparisons using the default Tukey adjustment for multiple comparisons, and it was used to calculate effect sizes.

Contrast	*df*	*Lower 95% confidence interval*	*Upper 95% confidence interval*	*Effect size (Cohen's d)*	*t*	*P*
E. cloacae – L. lactis	55	0.0405	0.0952	-6.40	-17.23	< 0.0001
E. cloacae – Pr. burhodogranariea	55	0.0748	0.1759	-4.99	-13.42	< 0.0001
E. cloacae – Ps. entomophila	55	0.0152	0.0358	-8.65	-23.29	< 0.0001
L. lactis – Pr. burhodogranariea	55	1.2146	2.8138	1.42	3.88	0.0016
L. lactis – Ps. entomophila	55	0.2472	0.5726	-2.25	-6.17	< 0.0001
Pr. burhodogranariea – Ps. entomophila	55	0.1337	0.3097	-3.67	-10.04	< 0.0001

Supplementary Table 2. The effect of bacterial species on virulence for smoothing parameters 1, 3 and 5 (Model 1, linear models, two-tailed). Bacterial species is included as a factor and the response variable is virulence, measured as the maximum hazard calculated separately for each dose and replicate, using smoothing parameters 1, 3 or 5. The results using smoothing parameter 2 are shown in the main text. For details see methods.

Smoothing parameter	*Tested effect*	*df*	*F*	*P*
1	Bacterial species	3,55	157.74	<0.0001
3	Bacterial species	3,55	203.17	<0.0001
5	Bacterial species	3,55	189.31	<0.0001

Supplementary Table 3a-d. Sample sizes (n) by bacterial species, days post injection and injection dose for Figure 4a-d, where n is the number of flies examined over three independent experiments.

Supplementary Table 3a - *E. cloacae* (Figure 4a)

Days post injection	*92 CFU*	*920 CFU*	*1,840 CFU*	*9,200 CFU*	*92,000 CFU*
1	10	10	10	10	10
2	10	10	10	10	10
3	10	10	10	10	10
4	10	10	10	10	10
7	10	10	10	10	10
14	10	10	10	10	10
21	10	10	10	10	10
28	10	10	10	10	10
35	10	10	10	10	9

Supplementary Table 3b - *Pr. burhodogranariea* (Figure 4b)

Days post injection	*92 CFU*	*920 CFU*	*1,840 CFU*	*9,200 CFU*	*92,000 CFU*
1	12	12	12	12	12
2	12	12	12	12	12
3	12	12	12	12	12
4	12	12	12	11	12
7	12	9	12	10	9
14	12	9	12	10	5
21	12	3	4	4	4
28	6	4	0	1	0
35	3	0	0	1	0

Supplementary Table 3c - *L. lactis* (Figure 4c)

Days post injection	*92 CFU*	*920 CFU*	*1,840 CFU*	*9,200 CFU*	*92,000 CFU*
1	8	8	8	8	8
2	12	12	12	12	12
3	12	12	12	12	11
4	12	12	12	12	4
7	12	12	12	10	0
14	12	8	5	5	0
21	11	0	1	0	0
28	2	0	0	0	0
35	0	0	0	0	0

Supplementary Table 3d - *Ps. entomophila* (Figure 4d)

Days post injection	*92 CFU*	*920 CFU*	*1,840 CFU*	*9,200 CFU*	*92,000 CFU*
1	8	8	7	5	5
2	12	8	3	2	1
3	2	5	1	1	0
4	0	2	0	0	0
7	0	0	0	0	0
14	0	0	0	0	0
21	0	0	0	0	0
28	0	0	0	0	0
35	0	0	0	0	0

Supplementary Table 4. Pairwise comparisons between bacterial species for differences in exploitation (infection intensity) (Model 3). The "emmeans" [4] statistical package in R was used to perform multiple comparisons using the default Tukey adjustment for multiple comparisons, and it was used to calculate effect sizes and confidence intervals for each comparison.

Contrast	*df*	*Lower 95% confidence interval*	*Upper 95% confidence interval*	*Effect size (Cohen's d)*	*t*	*P*
E. cloacae – L. lactis	35	0.000026	0.0031	-3.52	-8.36	< 0.0001
E. cloacae – Pr. burhodogranariea	35	0.001141	0.0854	-1.98	-5.24	< 0.0001
L. lactis – Pr. burhodogranariea	35	3.44	359.83	1.53	3.75	0.0018

Supplementary Table 5. The effect of initial injection dose on bacterial load at seven days post injection (Model 4, linear models, two-tailed). Experimental replicate and the person performing the injection were also included as factors in the models. *Ps. entomophila* was not analysed because it caused high fly mortality. * This model was run after excluding two flies that had been assigned the highest bacterial load value because their loads were over the detection limit.

Injected bacterium	*Tested effect*	*df*	*F*	*P*
E. cloacae	Log(Injection dose)	1,25	26.41	<0.0001
	Person	1,25	0.16	0.69
	Replicate	2,25	1.78	0.19
Pr. burhodogranariea	Log(Log(Injection dose))	1,45	66.60	<0.0001
	Person	1,45	2.58	0.087
	Replicate	2,45	0.36	0.55
L. lactis	Log(Injection dose)	1,37	3.81	0.058
	Person	1,37	0.71	0.40
	Replicate	2,37	1.98	0.15
*L. lactis**	Log(Injection dose)	1,35	4.59	0.039
	Person	1,35	2.49	0.12
	Replicate	2,35	1.43	0.25

Supplementary Table 6. The effect of log bacterial load (exploitation) and PPP on two different clearance indices (Model 6, generalised linear mixed models, two-tailed). Confidence intervals were calculated using the "confint" function in the "glmmTMB" [5] package based on the "profile" method.

Response variable	*Tested effect*	*df*	*Estimate*	*Lower 95% CI*	*Upper 95% CI*	*Effect size (Spearman's ρ)*	*Chisq*	*P*
Clearance $index_{3,4}$	Log (geometric mean bacterial load)	1	-0.790	-1.610	-0.550	-0.553	13.20	0.00028
	PPP	1	-1.226	-4.758	3.525	-0.360	0.43	0.512
Clearance $index_{7,14,21}$	Log (geometric mean bacterial load)	1	-0.579	-0.805	-0.397	-0.747	35.92	< 0.0001
	PPP	1	1.810	-5.042	8.477	-0.495	0.34	0.561

Supplementary Table 7. The effect of injection dose on presence/absence of infection in dead flies (Model 7, generalised linear model, two-tailed). Person performing the injection was also included as a factor in the models, and replicate was included for the analysis for *Pr. burhodogranariea* infections.

Injected bacterium	*Tested effect*	*df*	*LR Chisq*	*P*
E. cloacae	Log(Log(Injection dose))	1	4.71	0.030
	Log(Day post injection)	1	0.13	0.72
	Person	1	1.11	0.29
	Log(Log(Injection dose)) × Log(Day post injection)	1	0.11	0.74
Pr. burhodogranariea	Log(Log(Injection dose))	1	13.45	0.00024
	Log(Day post injection)	1	3.48	0.062
	Person	1	5.02	0.025
	Replicate	1	0.48	0.49
	Log(Log(Injection dose)) × Log(Day post injection)	1	2.08	0.15

References

1. Råberg, L. and M. Stjernman, *The evolutionary ecology of infectious disease virulence*, in *Ecological Immunology*, G. Demas and R. Nelson, Editors. 2012, Oxford University Press. p. 548-578.
2. Best, A., A. White, and M. Boots, *Maintenance of host variation in tolerance to pathogens and parasites.* Proceedings of the National Academy of Sciences, 2008. 105(52): p. 20786-20791.
3. Råberg, L., A.L. Graham, and A.F. Read, *Decomposing health: tolerance and resistance to parasites in animals.* Philosophical Transactions of the Royal Society B: Biological Sciences, 2009. 364(1513): p. 37-49.
4. Lenth, R., *emmeans: Estimated marginal means, aka least-square means.* 2020.
5. Brooks, M.E., et al., *glmmTMB balances speed and flexibility among packages for zero-inflated generalized linear mixed modeling.* The R journal, 2017. 9(2): p. 378-400.
