# Supplementary material for: Decomposing virulence to understand bacterial clearance in persistent infections
Source: Nat Commun. 2022 Aug 26;13:5023. doi: 10.1038/s41467-022-32118-1 (PMC9418333; doi:10.1038/s41467-022-32118-1)
Supplement: Supplementary file 3 — Reporting Summary [file 41467_2022_32118_MOESM3_ESM.pdf]

Corresponding author(s): Sophie A.O. Armitage

Last updated by author(s): Jul 7, 2022

Reporting Summary

Nature Portfolio wishes to improve the reproducibility of the work that we publish. This form provides structure for consistency and transparency in reporting. For further information on Nature Portfolio policies, see our Editorial Policies and the Editorial Policy Checklist.

Statistics

For all statistical analyses, confirm that the following items are present in the figure legend, table legend, main text, or Methods section.

n/a	Confirmed	
☐	☒	The exact sample size (n) for each experimental group/condition, given as a discrete number and unit of measurement
☐	☒	A statement on whether measurements were taken from distinct samples or whether the same sample was measured repeatedly
☐	☒	The statistical test(s) used AND whether they are one- or two-sided<br>*Only common tests should be described solely by name; describe more complex techniques in the Methods section.*
☐	☒	A description of all covariates tested
☐	☒	A description of any assumptions or corrections, such as tests of normality and adjustment for multiple comparisons
☐	☒	A full description of the statistical parameters including central tendency (e.g. means) or other basic estimates (e.g. regression coefficient) AND variation (e.g. standard deviation) or associated estimates of uncertainty (e.g. confidence intervals)
☐	☒	For null hypothesis testing, the test statistic (e.g. F, t, r) with confidence intervals, effect sizes, degrees of freedom and P value noted<br>*Give P values as exact values whenever suitable.*
☒	☐	For Bayesian analysis, information on the choice of priors and Markov chain Monte Carlo settings
☒	☐	For hierarchical and complex designs, identification of the appropriate level for tests and full reporting of outcomes
☐	☒	Estimates of effect sizes (e.g. Cohen's d, Pearson's r), indicating how they were calculated

Our web collection on statistics for biologists contains articles on many of the points above.

Software and code

Policy information about availability of computer code

Data collection	No software was used.
Data analysis	As stated in the Methods section and the Supplementary Methods, all data analyses were performed in RStudio, version 1.3.1073. Details of the statistical models that we tested can be found in the supplementary methods. The packages that we used for statistics and for plotting the data within RStudio are: car (v 3.1.0), dplyr (v 1.0.9), DHARMa (v 0.4.5), emmeans (v 1.7.5), EnvStats (v 2.7.0), ggplot2 (v 3.3.6), ggpubr (v 0.4.0), glmmTMB (v 1.1.3), gridExtra (v 2.3), magrittr (v.2.0.3), muhaz (v 1.2.6.4), plyr (v 1.8.7), psych (v 2.2.5), purrr (v 0.3.4), scales (v. 1.2.0), survival (v 3.3.1), survminer (v 0.4.9), tidyr (v 1.2.0) and viridis (v 0.6.2). Code used to create the figures and for the analyses is publicly available on Refubium, the institutional repository of the Freie Universitaet Berlin: http://dx.doi.org/10.17169/refubium-35174.

For manuscripts utilizing custom algorithms or software that are central to the research but not yet described in published literature, software must be made available to editors and reviewers. We strongly encourage code deposition in a community repository (e.g. GitHub). See the Nature Portfolio guidelines for submitting code & software for further information.

Data

Policy information about availability of data

All manuscripts must include a data availability statement. This statement should provide the following information, where applicable:

- Accession codes, unique identifiers, or web links for publicly available datasets
- A description of any restrictions on data availability
- For clinical datasets or third party data, please ensure that the statement adheres to our policy

The data are publicly available on Refubium, the institutional repository of the Freie Universitaet Berlin: http://dx.doi.org/10.17169/refubium-35174

Field-specific reporting

Please select the one below that is the best fit for your research. If you are not sure, read the appropriate sections before making your selection.

☐ Life sciences ☐ Behavioural & social sciences ☒ Ecological, evolutionary & environmental sciences

For a reference copy of the document with all sections, see nature.com/documents/nr-reporting-summary-flat.pdf

Ecological, evolutionary & environmental sciences study design

All studies must disclose on these points even when the disclosure is negative.

Study description	The study has a fully factorial design. Flies were infected with one of four different bacterial species. For infection with each bacterial species, we performed three independent experiments (i.e., three experimental replicates). For each experimental replicate we had seven treatment groups: injection with one of five bacterial concentrations, an injection control (Ringer's injected) and a naïve group that were not injected. For each experimental replicate we treated 252 flies, giving a total of 756 flies per bacterial species (including Ringer's and naïve flies). Per experimental replicate and treatment, 36 flies were checked daily for survival until all flies were dead. To evaluate bacterial load in living flies, four flies per treatment per replicate were homogenised for each of nine different time points. Some of the bacterial species were particularly virulent, and due to high mortality, we were not able to assay four flies for all of the time points for all of the doses. We also homogenised flies that had died within 24 hours of death, in order to determine whether they had died whilst being infected. This was done at between 14- and 35-days and 56- and 78-days post injection. The first-time frame covers part of the time frame for living flies, and the second time frame was to give us a longer-term estimate of infection persistence.
Research sample	The research sample that we used is meant to represent an outbred wild-type population of Drosophila melanogaster. In more detail, we used an outbred stock population of Drosophila melanogaster established from 160 Wolbachia-infected fertilised females collected in Azeitão, Portugal (https://doi.org/10.1371/journal.ppat.1003601) and given to us by Élio Sucena. We used female flies, as our previous work on this topic has focussed on this sex. The flies were reared at a standardised density for one generation prior to experimental infection, and they were infected when they had been in the adult stage for four days. They were given access to males up to day four, but they were kept in single sex groups after infection.
Sampling strategy	For the survival experiments we used between 79-108 flies per treatment group. These numbers of individuals were used because they are in the range of those used per treatment group in other studies on a similar topic, which we also cite in our paper, e.g., Chambers et al. 2019 (n = 80 to 134 individuals in Fig. 1; https://doi.org/10.1371/journal.pone.0224440), Duneau et al. 2017 (n = between 20 and 80 individuals; https://doi.org/10.7554/eLife.28298.001).<br><br>For the bacterial load, we predicted that differences between treatment groups would be visible despite high amounts of variation, using relatively low sample sizes. We therefore assayed the maximum number of flies that could be reliably processed. The samples sizes that we used per treatment are similar to those used by other studies published on a similar topic and which we cite in our paper, e.g., Haine et al. 2008 (n = 7 to 10 individuals; 10.1126/science.1165265), Louie et al. 2016 (n = at least six individuals; doi:10.1371/journal.pbio.1002435), Chambers et al. 2019 (n = six or more individuals; https://doi.org/10.1371/journal.pone.0224440), except where there was high mortality at later infection time points/intermediate to high virulence bacteria.
Data collection	The experiments were set up so that two people performed all injections and homogenisations in a fully factorial manner. The survival checks were performed by three people. The data were recorded on paper, which was later transferred to Excel. The correct entry of all data into Excel was double-checked.
Timing and spatial scale	The experiments were performed between November 2018 and June 2019. More specifically the injection dates were as follows: Enterobacter cloacae: 10.11.2018, 25.3.2019, 8.4.2019 (note that we also carried out injections with E. cloacae on two other dates, but that these experimental replicates were abandoned due to contamination in the samples); Lactococcus lacis: 7.11.2018, 12.11.2018, 11.2.2019; Providencia burhodogranariea: 8.11.2018, 13.11.2018, 4.2.3019; Pseudmonas entomophila: 9.11.2018, 14.11.2018, 8.1.2019. Survival was checked daily until all flies were dead. Daily survival checks are typically performed in this kind of eco-evo-immunity experiment. Bacterial load was assayed at 1, 2, 3, 4, 7, 14, 21, 28 and 35 days after infection in order to provide a short- and longer-term idea about infection load. We sampled bacterial load at time points that were closer together for the first few days because this is where the most variation was anticipated in the bacterial load. There is no spatial scale for the experiments.
Data exclusions	As stated in the materials and methods, out of 1223 flies homogenised, bacterial load data from 27 flies were excluded, given that contamination made counts of the injected bacteria unreliable. Exclusion criteria were established as a result of this contamination. Furthermore, we provide the results from one statistical test both with and without two flies, given that they had too many bacterial colony forming units to be able to accurately estimate the load. This point is also stated in the results.
Reproducibility	Infections with all four bacterial species were replicated independently three times. Two additional replicates that had been infected with E. cloacae were not utilised because of technical problems with contamination.
Randomization	On any one day when an experiment was being set up, there were seven treatments: five different doses of bacteria, one naïve group and one Ringer's injected (injection control) group. Flies were randomly allocated to one of these seven groups by previously randomising the treatment order using random.org. A different randomised order was used for each experimental replicate.
Blinding	Blinding per bacteria species was not possible given that one bacterial species was assayed per experiment. We used numbering on the vials containing the flies instead of treatment groups, so that we did not know what treatment group the flies belonged to by looking at the vials. Bacterial loads were assayed blind with respect to treatment.

Did the study involve field work? ☐ Yes ☒ No

Reporting for specific materials, systems and methods

We require information from authors about some types of materials, experimental systems and methods used in many studies. Here, indicate whether each material, system or method listed is relevant to your study. If you are not sure if a list item applies to your research, read the appropriate section before selecting a response.

Materials & experimental systems

n/a	Involved in the study
☒	☐ Antibodies
☒	☐ Eukaryotic cell lines
☒	☐ Palaeontology and archaeology
☐	☒ Animals and other organisms
☒	☐ Human research participants
☒	☐ Clinical data
☒	☐ Dual use research of concern

Methods

n/a	Involved in the study
☒	☐ ChIP-seq
☒	☐ Flow cytometry
☒	☐ MRI-based neuroimaging

Animals and other organisms

Policy information about studies involving animals; ARRIVE guidelines recommended for reporting animal research

Laboratory animals	We used an outbred stock population of Drosophila melanogaster established from 160 Wolbachia-infected fertilised females collected in Azeitão, Portugal (described here: https://doi.org/10.1371/journal.ppat.1003601) and given to us by Élio Sucena (Gulbenkian Institute, Portugal). We used female flies, previously allowed access to males, and which were four-days post adult eclosion at the time of infection. Note that we used an outbred wild-type population, and that this population does not have a strain name.
Wild animals	The study did not involve wild animals
Field-collected samples	The study did not involve samples collected from the field
Ethics oversight	No ethical approval or guidance was required given that the model host was the insect Drosophila melanogaster. We injected the flies with microorganisms that pose no threat to healthy people or the environment.

Note that full information on the approval of the study protocol must also be provided in the manuscript.
